# Early Activation of Rat Skeletal Muscle IL-6/STAT1/STAT3 Dependent Gene Expression in Resistance Exercise Linked to Hypertrophy

**DOI:** 10.1371/journal.pone.0057141

**Published:** 2013-02-22

**Authors:** Gwénaëlle Begue, Aymeric Douillard, Olivier Galbes, Bernadette Rossano, Barbara Vernus, Robin Candau, Guillaume Py

**Affiliations:** INRA, UMR866 Dynamique Musculaire et Métabolisme, Université Montpellier 1, F-34060, Montpellier, France; Ohio State University, United States of America

## Abstract

Cytokine interleukin-6 (IL-6) is an essential regulator of satellite cell-mediated hypertrophic muscle growth through the transcription factor signal transducer and activator of transcription 3 (STAT3). The importance of this pathway linked to the modulation of myogenic regulatory factors expression in rat skeletal muscle undergoing hypertrophy following resistance exercise, has not been investigated. In this study, the phosphorylation and nuclear localization of STAT3, together with IL-6/STAT3-responsive gene expression, were measured after both a single bout of resistance exercise and 10 weeks of training. Flexor Digitorum Profundus muscle samples from Wistar rats were obtained 2 and 6 hours after a single bout of resistance exercise and 72 h after the last bout of either 2, 4, or 10 weeks of resistance training. We observed an increase in IL-6 and SOCS3 mRNAs concomitant with phosphorylation of STAT1 and STAT3 after 2 and 6 hours of a single bout of exercise (p<0.05). STAT3-dependent early responsive genes such as CyclinD1 and cMyc were also upregulated whereas MyoD and Myf5 mRNAs were downregulated (p<0.05). BrdU-positive satellite cells increased at 2 and 6 hours after exercise (p<0.05). Muscle fiber hypertrophy reached up to 100% after 10 weeks of training and the mRNA expression of Myf5, c-Myc and Cyclin-D1 decreased, whereas IL-6 mRNA remained upregulated. We conclude that the IL-6/STAT1/STAT3 signaling pathway and its responsive genes after a single bout of resistance exercise are an important event regulating the SC pool and behavior involved in muscle hypertrophy after ten weeks of training in rat skeletal muscle.

## Introduction

Human strength training is well known to increase skeletal muscle mass and induce muscle phenotypic changes [1]. Increase in muscle strength resulting from skeletal muscle hypertrophy is of great interest to people including elite power athletes, patients rehabilitating from disease-induced atrophy and the elderly who have diminished mobility due to muscular weakness. Muscle hypertrophy is induced by cellular and molecular mechanisms including a number of signaling pathways leading to an increase in protein synthesis and a decrease in protein breakdown.

Skeletal muscle satellite cells (SCs) are a group of quiescent cells located between the basal lamina and plasma membrane of the myofibers in mature muscles [2]. These cells are mainly responsible for postnatal muscle growth by hypertrophy [3], [4] as well as for exercise- or injury-induced muscle regeneration [5]. Indeed, resistance/strength training can increase SC activity and/or the number of myonuclei [6], [7]. Although SCs are key regulators of muscle growth during development and muscle adaptation following exercise [8]–[10], the cellular regulation of the SC function remains largely unexplored.

Recently, interleukin-6 (IL-6) has been implicated as part of the activation of human SCs in response to damaging eccentric contractions [11], [12]. Traditionally, IL-6 is considered as a pleiotropic pro-inflammatory cytokine associated with the control and coordination of immune responses [13]. Increasing evidence indicates that skeletal muscle cells are an additional important source of IL-6 after a single bout of endurance exercise in humans or overload induced hypertrophy in rodent [14]–[16], at least in part under the dependence of the serum responsible factor (SRF) [17]. Interestingly, IL-6 knock-out (IL-6^−^/^−^) mice demonstrated a blunted hypertrophic response and a lower SC-related myonuclear accretion compared to wild-type mice following compensatory hypertrophy [18]. Furthermore, SC from IL-6^−^/^−^ mice demonstrated an impaired proliferative capacity, both in vivo and in vitro. This impairment was related to a lack of IL-6 mediated activation of signal transducer and activator of transcription-3 (STAT3) signaling. The activation of Janus tyrosine kinases (JAKs) by IL-6 leads to STAT3 phosphorylation (pSTAT3) and activation which elicits dimerization and translocation of pSTAT3 into nucleus [19]–[21]. pSTAT3 induces the transcription of downstream genes involved in several biological functions [22] including cell proliferation, differentiation, and survival of myoblasts. These responses are mediated by the expression of cell cycle regulators *c-myc* and *cyclinD1* [23], [24], the antiapoptotic genes *Bcl-2* and *Bcl-xL* [25]–[27] and intermediate early response genes such as *c-fos* and *junB* [28], as well as the angiogenic factor (*VEGF*) [29] and the suppressor of cytokine signaling 3 *(SOCS3)* [19]. Moreover, Yang *et al.* (2009) reported that STAT3 could interact with MyoD, the STAT3-MyoD complex being responsible for the stimulatory effect of STAT3 on myogenic differentiation [30].

During recovery from exercise, the activation of STAT3 signaling has been demonstrated in human skeletal muscle [20], [31]. However, few studies have explored the link between the IL-6/JAK/STAT pathway and SC behavior in the muscular hypertrophy induced by strength or resistance training both in animals or humans. For example, whether or not the muscle IL-6 response still persists after several weeks of training has not yet been investigated. Moreover, the precise mechanisms of the IL-6/JAK/STAT pathway on satellite cell behavior via the regulation of the known myogenic regulatory factors have to be defined in resistance training-induced skeletal muscle hypertrophy. For that purpose, we modified a physiological exercise model from Lee *et al.* (2004) [32] to investigate the underlying molecular and cellular events related to the IL-6/JAK/STAT3 pathway of the rat forearm limb muscle Flexor Digitorum Profundus (FDP) after either a single bout of exercise or after 2, 4 and 10 weeks of voluntary resistance training. We hypothesized that 10 weeks of intense resistance training would lead to hypertrophy linked to repeated muscle IL-6/STAT3-dependent gene stimulation, particularly those genes related to satellite cell behavior, after each single bout of resistance exercise [33].

## Materials and Methods

### Ethics statement

This study was approved by the Committee on the Ethics of Animal Experiments of the Languedoc Roussillon in accordance with the guidelines from the French National Research Council for the Care and Use of Laboratory Animals. (Permit Number: CEEA-LR-1069). All surgery was performed under sodium pentobarbital anesthesia, and all efforts were made to minimize suffering.

### Animals

48 Male Wistar Han rats, weighing around 220 g, were purchased from Charles River (Charles River Laboratories International, Wilmington, MA) and housed at a constant room temperature and humidity and maintained at a 12:12 h light-dark cycle. Rats had access to standard rat chow and water *ad libitum*.

### Experimental design

Rats were exercised in apparatus adapted from Lee *et al.* (2004) [32]. A 1-m ladder with 2-cm grid steps and inclined at 85° was made in our laboratory. Initially, rats were familiarized with the ladder by practicing voluntary climbing of the ladder from the bottom to the top cage for one week, after which the strength-training or exercise regimen started. Cloth bags containing weights were attached to the base of the tail with a Velcro strap.

#### Resistance training Protocol

After one week of adaptation, 36 rats were randomly divided into six groups: CTL2, CTL4, CTL10 (CTL = non-training controls, n = 6 in each group) and TR2, TR4 and TR10, which were rats trained for 2, 4 and 10 weeks (Training Resistance n = 6 in each group). The initial weight attached to the tail of each animal was 50% of its body weight (bw). Rats were positioned at the bottom of the climbing apparatus and when they reached the top of the ladder, they were allowed to rest in a simulated home cage for 2 min. Rats performed 10 repetitions or climbs, five times a week during 2 (TR2), 4 (TR4) or 10 (TR10) weeks. Training was performed every afternoon. Loads were increased by 10% every 2 days but only if the rat was able to perform 10 climbs per set. After 2 weeks of training, the load reached 120% of bw, 150% after 4 weeks and 210% after 10 weeks of training. Maximal repetition was determined as the maximum weight carried up the exercise ladder by the rats in one climb and was only measured on the last day after 10 weeks training. 72 hours after the last training bout, rats were killed via an intraperitoneal injection of pentobarbital 50 mg.kg^−1^ (Penthotal®). The forearm muscle, Flexor Digitorum Profundus (FDP), was dissected, frozen in isopentane chilled in liquid nitrogen and stored at −80°C for later use.

#### Single resistance exercise protocol

After one week of adaptation, rats were randomly divided into three groups: REST (n = 4) with rats sacrificed just before exercise, E2H (n = 4) and E6H (n = 4) where rats were sacrificed 2 and 6 hours after the single bout of exercise respectively. Twenty-four hours before sacrifice, the animals of each group were injected intraperitoneally with 100 mg.kg^−1^ of bromodeoxyuridine (BrdU, B5002, Sigma Aldrich, St Louis, MO) to identify cells in proliferation. In the afternoon, rats of the E2H and E6H groups made 4 climbs with a load that reached 25% of bw, 4 climbs at 50%, 4 climbs at 75% and 6 climbs at 100% of bw. Between each climb, rats were allowed to rest for 2 min. Rats were anesthetized via an intraperitoneal injection of pentobarbital 50 mg.kg^−1^ (Penthotal®). The forearm FDP muscles were harvested, frozen in isopentane chilled in liquid nitrogen and stored at −80°C for later use. The animals were then killed by an overdose of pentobarbital.

### Myosin Heavy Chain Immunohistochemistry

For fiber type analysis, transverse serial sections of FDP muscles (10 µm thick) were obtained using a cryostat at −20°C. Frozen sections were fixed with acetone solution for 10 min, washed and incubated 30 min in phosphate buffered saline (PBS) blocking solution with 2% bovine serum albumin (BSA). Sections were then incubated 2 hours at room temperature with mouse monoclonal antibody (DSHB, Iowa City, Iowa) directed against MHC-I (#A4-971), MHC IIa (#2F7), MHC IIx (#6H1) and MHC-IIb (#10F5). Sections were then washed three times with PBS and incubated one hour at 37°C with peroxidase-conjugated rabbit anti-mouse IgG secondary antibody (A-9044, Sigma-Aldrich, St Louis, MO). MHC staining was revealed with NovaRed™ (Vector® Lab, Burlingame, CA) and slides mounted with Mowiol. Images were captured with a microscope (ZEISS Axiophot) coupled with a CCD camera connected to a computer. MHC analysis was realized with Image-J® software. Muscle fiber Cross-Sectional Area (CSA) was obtained in ×10 magnification images from 1500 fibers per group (250 fibers per muscle from 6 rats).

### Western Blotting

20 mg FDP samples were homogenized in 10 volumes of lysis buffer (Tris 20 mM pH 6,8, NaCl 100 mM, EGTA 1 mM, NaF 100 mM, Triton X-100 0,5%, Na_3_VO_4_ 5 mM) with inhibitory protease cocktails (P8340, Sigma Aldrich, St Louis, MO). The homogenate was rotated 10 min at 4°C and the supernatant collected. Protein samples (50 µg) were denatured and separated on 10% SDS-PAGE. The proteins were transferred onto a nitrocellulose membrane and blocked in 5% wt/vol BSA for phosphoSTAT1 (Tyr 701), phosphoSTAT3 (Tyr 705), phosphoErk1/2 (Tyr 202/204) and 5% wt/vol dry milk for STAT1, STAT3, Erk1/2 and α-tubulin antibodies in Tris-buffered saline with 0.1% vol/vol Tween 20 (TBST) for one hour at room temperature. Primary antibodies pSTAT1 (1/1000), pSTAT3 (1/1000), pErk1/2 (1/1000), STAT1 (1/1000), STAT3 (1/1000), Erk1/2 (1/1000) and α-tubulin (1/2500) diluted in blocking buffer were applied and incubated overnight at 4°C. Membranes were subsequently washed three times with TBST and incubated one hour at room temperature with a secondary antibody conjugated to horseradish peroxidase, donkey anti-rabbit IgG (1/4000) for pSTAT1, pSTAT3, Erk1/2, STAT1 and STAT3 and rabbit anti-mouse IgG (1/4000) for pErk1/2 and α-tubulin. Proteins were visualized by enhanced chemiluminescence (32106, Pierce®, Rockford, IL) and quantified with ImageJ® software. Levels of pSTAT1, pSTAT3 and pErk1/2 proteins were expressed relative to total STAT1, STAT3, Erk1/2 respectively. Antibodies pSTAT1 (#9171), pSTAT3 (#9131), pErk1/2 (#9106), STAT1 (#9172), STAT3 (#9132) and Erk1/2 (#9102) were purchased from Cell Signaling Technology (Danvers, MA), α-tubulin (#T6199) and secondary antibody rabbit anti-mouse (#A9044) from Sigma-Aldrich (St Louis, MO) and secondary antibody donkey anti-rabbit (#NA934V) from GE Healthcare (Buckinghamshire, UK).

### pSTAT3, Pax7 and BrdU immunohistochemistry

12 µm thick FDP muscle sections were either co-stained for pSTAT3 and Pax7 antibodies to distinguish the cellular localization of STAT3 activation in nuclei of myocytes or in nuclei of both quiescent and activated satellite cells (Pax7^+^ cell) or co-stained for BrdU and laminin antibodies to assess SCs mitotic activity. All cryosections were rehydrated in phosphate buffered saline (PBS). For pSTAT3 and Pax7 staining, sections were blocked in 1% BSA for one hour at room temperature. Primary antibodies were incubated with 0.1% Triton X-100 and 1% BSA overnight at 4°C. PBS was used for all washing steps throughout staining, with the exception of Pax7, where Tris-buffered saline (TBS) was used to wash the sections. Muscle sections were first stained for pSTAT3 and followed by a second staining for Pax7 (DSHB, Iowa City, Iowa). After incubation with pSTAT3 primary antibody (1/50), the sections were washed in PBS, and goat anti-rabbit IgG secondary antibody (1/1000, Alexa Fluor® 488, #A-11034, Invitrogen Life Technologies, Renfrew, UK) was applied for 30 min at 37°C, post-fixed in 1% paraformaldehyde (PAF) for 10 min, incubated with the second primary antibody Pax7 (undiluted) overnight at 4°C. Sections were washed in TBS and goat anti-mouse IgG secondary antibody (1/1000; Alexa Fluor® 568, #A-11031, Invitrogen Life technologies, Renfrew, UK) was applied for 30 min at 37°C, post-stained with Hoescht and fixed in 1% PAF. For BrdU and laminin labeling, sections were fixed in 4% PAF (pH 7.2) for 5 min, washed in PBS and placed in 2M HCl at 56°C for 30 min in order to denaturate double-stranded DNA. After neutralization with 0.1M sodium borate (pH 8.5) for 10 min, sections were washed in PBS and blocked with normal goat serum (1/30, G9023, Sigma) for 1 h at 25°C. Muscle sections were first stained for BrdU and followed by a second staining for laminin. After incubation in monoclonal BrdU antibody (1/50, B-2531, Sigma) in PBS with 0.2% BSA and 0.05% Tween-20 for 2 h at 25°C, the sections were washed in PBS and goat anti-mouse Alexa Fluor® 568 secondary antibody (1/250) was applied for 1H30 at 25°C. Sections were post-fixed in 4% PAF and incubated with second primary polyclonal antibody laminin (1/50, L9393, Sigma) diluted in 1% BSA for 30 min at 37°C. After washing, goat anti-rabbit Alexa Fluor® 488 secondary antibody (1/1000) was applied on sections for 30 min at 37°C. Sections were washed and post-fixed in 4% PAF. All sections were mounted with Dako Fluorescent Mounting Medium (Dako, #S3023, Carpinteria, CA) and images were collected using a microscope (ZEISS Axiophot) coupled with a CCD camera and analyzed using ImageJ® software. BrdU-stained cells were counted as SCs when located intra-laminin staining and correlated to the number of fibers (∼500 fibers per muscle).

### RNA Extraction and Real Time Polymerase Chain Reaction

RNA was isolated from homogenate muscle samples using the RNeasy mini Kit following the manufacturer's instructions (Qiagen, Germantown, MD). The RNA was quantified with a spectrophotometer (Eppendorf AG, Hamburg, Germany). RNA integrity was electrophoretically verified by ethidium bromide staining and by OD_260_/OD_280_ nm absorption ratio >1.95. 2 µg RNA of each sample was reverse transcribed to cDNA in 20 µl reactions using a commercially available kit (High Capacity cDNA Reverse Transcription Kit; Applied Biosystem Life Technologies Carlsbad, CA) according to the manufacturer's instructions. The cDNA synthesis reaction was carried out using thermal cycler (MiniCycler™, MJ Research, St Bruno, Canada) and followed by 10 times dilution with ultra-pure water containing denaturated salmon sperm DNA. Forward (F) and Reverse (R) primers used to amplify genes are listed in Table 1. Quantitative real-time PCR was performed in a 20 µl final volume with 250 nM of each primer using iQ SYBR Green Supermix (Bio-rad, Hercules, CA). After incubation at 95°C for 10 min, the cycling protocol was performed in MiniOpticon™ (Bio-rad) as follows for IL-6, LIF, SOCS3 and cMyc: 10 s at 95°C for denaturation, 30 s at 60°C for annealing. For the reaction of Myogenin, the annealing temperature was set at 61°C, for Rpl32, CycloA and CyclinD1 at 63°C and for MyoD, Myf5 and Pax7 at 64°C. After 40 cycles of PCR, melting curve analysis was performed to check primer specificity. All Cq values were analyzed using a comparative critical threshold method previously described by Pfaffl (2001) [34]. Transcription levels were normalized using Cq arithmetic mean of two reference genes: CyclophilinA and Rpl32.

**Table 1:** PCR Primer set sequences.

Gene	Forward (5′-3′)	Reverse (5′-3′)	NCBI (Reference Sequence)	Amplicon Size, bp
IL6^a^	TGTATGAACAGCGATGATG	AGAAGACCAGAGCAGATT	NM_012589.1	128
LIF^b^	ACCAGATCAAGAGTCAACTG	CCTTGAGCTGTGTAATAGGA	NM_022196.2	76
Ppia^c^	TTTGGGAAGGTGAAAGAAGGC	CACAGTCGGAGATGGTGAT	NM_017101.1	100
Rpl32^d^	TCTGGTCCACAATGTCAAGG	CTGCTCTTTCTACGATGGCT	NM_013226.2	116
Myc^e^	GAAGAACAAGATGATGAGGAA	GCTGGTGAGTAGAGACAT	NM_012603.2	160
Ccnd1^f^	AGATGAAGGAGACCATTCCC	GCCAGGTTCCATTTGAGC	NM_171992.4	124
Socs3^g^	GATCCCGCTGGTACTGA	TGACCGTTGACAGTCTTC	NM_053565.1	81
Myf5^h^	GGAATGCAATCCGCTACATT	CAGGGCAGTAGATGCTGTCA	NM_001106783.1	192
MyoD1^i^	GGAGACATCCTCAAGCGATGC	GCACCTGGTAAATCGGATTG	NM_176079.1	104
Pax7^j^	GCCCTCAGTGAGTTCGATTAGC	TCCTTCCTCATCGTCCTCTTTC	NM_001191984	70
Myog^k^	AACCCAGGAGATCATTTGC	GGAAGGTGACAGACATATCC	NM_017115.2	109

a IL-6, interleukin 6;

b LIF, Leukemia Inhibitory Factor;

c Ppia, peptidylprolyl isomerase A (cyclophilin A);

d Rpl32, ribosomal protein L32;

e myc, myelocytomatosis oncogene;

f Ccnd1, CyclinD1;

g Socs3, suppressor of cytokine signaling 3;

h Myf5, myogenic factor 5;

i MyoD1, myogenic differentiation 1;

j Pax7, paired box 7;

k Myog, myogenin.

### Statistical analysis

All values are expressed as means ± SEM. A one-way ANOVA was employed to compare data. When a significant effect was indicated, a Fisher significant difference post hoc test was performed. Significance was set at p<0.05.

## Results

### Resistance training induces phenotypic changes and fiber hypertrophy of FDP muscle

This resistance training protocol induced an alteration in fiber type composition with marked changes from 4 weeks of training (TR4). The proportion of type I and IIx fibers decreased whereas that of IIa increased by 47% in TR4 and TR10 groups (Table 2). To confirm that the voluntary resistance training protocol could promote muscular hypertrophy, fiber cross-sectional areas (µm^2^) were measured at different time points after 2 (TR2), 4 (TR4) or 10 (TR10) weeks of training. Two and four weeks of training caused significant hypertrophy of type IIx, respectively +49% and +88% compared to respective control group (n = 6; p<0.05). As all FDP muscle fiber types were hypertrophied after 10 weeks of resistance training (TR10), we choose to focus all further analyses at this point in time (Table 2).

**Table 2:** Typology, mean fiber cross-sectional area and fiber area/nucleus ratio of FDP muscle after 2, 4 and 10 weeks of resistance training (TR2, TR4 and TR10 respectively).

		CTL2	TR2	CTL4	TR4	CTL10	TR10
Fiber Type Proportion (%)	Type I	24,29±4,76	12,27±3,47*	13,95±1,99	6,90±2,01*	13,76±2,09	3,94±1,63*
	Type IIa	38,62±5,97	36,53±3,17	22,37±2,24	47,10±4,97*	26,27±2,26	55,41±2,57*
	Type IIx	37,09±5,42	50,27±3,06	61,95±2,67	45,91±3,10*	59,85±3,09	40,65±3,54*
Cross-sectional area (µm^2^)	Type I	708,37±64,48	959,13±117,29	825,76±139,60	829,2±46,15	726,31±34,30	1287,33*±58,92
	Type IIa	945,5±6,9	1151,3±100,9	1049,3±55	1325,6*±98,6	1099,6±39,1	2109,1*±169,1
	Type IIx	1209,3±142,4	2271,8*±104,9	1762,8±84	2622,4*±236,3	2069,1±224,3	4138,5*±238,6
Myonuclear domain^a^		NM^b^	NM^b^	NM^b^	NM^b^	16,1±1,2	15,6±1,2

a Myonuclear domain is defined as the fiber area (µm^2^)/myonucleus number ratio obtained from 2000 fibers per group.

* Significantly different between *Training Resistance* (TR) and *Control* (CTL) groups (p<0.05).

b NM : not measured.

### 10 weeks of resistance training did not affect the myonuclear domain

We found that the increased cross-sectional area of muscle fiber after 10 weeks of training was not accompanied by any variation in the myonuclear domain value suggesting incorporation of new nuclei into the fibers (Table 2). Indeed, we noted a significant increase in the number of myonuclei per fiber cross-section (data not shown).

### STAT1 and STAT3 are phosphorylated in rat skeletal muscle following acute resistance exercise

**Figure 1:**
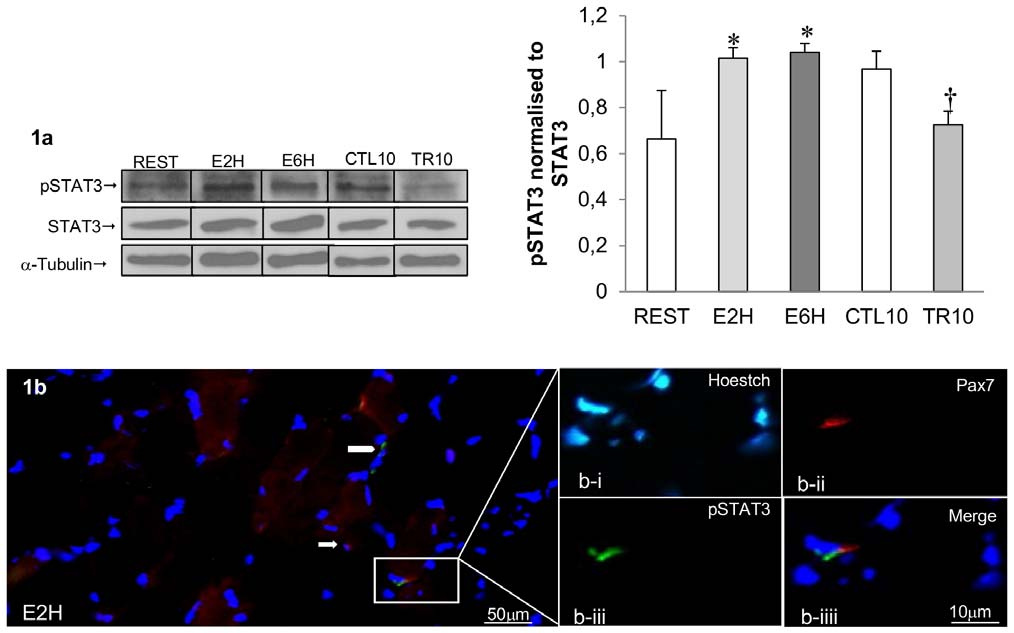
STAT3 activity in rat skeletal muscle following acute resistance exercise. (**1a**) Representative western blot from FDP muscle protein samples taken at REST, 2 hours (E2H), 6 hours (E6H) post-exercise and after 10 weeks of training (CTL10, TR10), with anti-phospho-STAT3 (Tyr705) (pSTAT3), anti-total STAT3 (tSTAT3) and anti-α-tubulin. The arrow indicates the pSTAT3 band at 79 kDa and α-tubulin at 55 kDa. Graph shows arbitrary units of pSTAT3 normalized to tSTAT3 representing the mean ± SEM of 4–6 rats * significantly different from REST (p<0.05); † significantly different from CTL10 (p<0.01). (**1b**) Representative merged image of E2H at 40× magnification with inset box showing (**b-i**) nuclei (Hoestch = blue), (**b-ii**) satellite cells (red = Pax7^+^ indicated by arrow), (**b-iii**) pSTAT3 protein (green = pSTAT3 indicated by arrowhead), (**b-iiii**) Pax7^+^/pSTAT3^+^ cell at 2 h (E2H). Note: No Pax7^+^/pSTAT3^+^ staining found at REST, E6H, CTL10 or TR10 (not shown).

**Figure 2:**
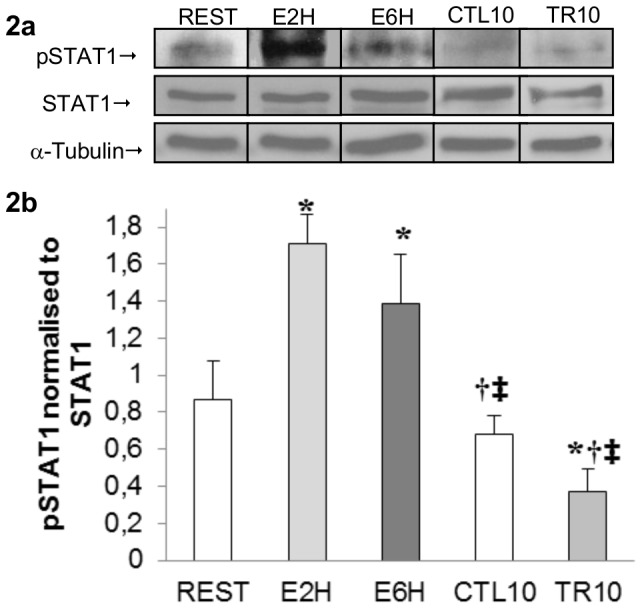
STAT1 activity in rat skeletal muscle following acute resistance exercise. ***A***: representative western blot of protein extracted from FDP muscle samples taken at REST, 2 hours (E2H), 6 hours (E6H) post-exercise and after 10 weeks of training (CTL10, TR10), with anti-phospho-STAT1 (Tyr701) (pSTAT1), anti-total STAT1 (tSTAT1) and anti-α-tubulin. The arrow indicates the pSTAT1 band at 91 kDa. and α-tubulin at 55 kDa. ***B***: The graph shows arbitrary units of pSTAT1 normalized to tSTAT1 representing the mean ± SEM of 4–6 rats. * Significantly different from resting value (REST; p<0.05). † Significantly different from E2H (p<0.01). ‡ Significantly different from E6H (p<0.01).

The activation of STAT1 and STAT3 were assessed by pSTAT1 tyrosine 701 and pSTAT3 tyrosine 705 in FDP muscle samples at REST, 2 hours (E2H) and 6 hours (E6H) after a single bout of resistance exercise as well as after 10 weeks of resistance training (TR10). 2 and 6 hours post-exercise, pSTAT3 (n = 4, p<0.01, Fig. 1*a*) and pSTAT1 had increased significantly (n = 4, p<0.05, Fig. 2) from resting value (REST). To confirm that the increased phosphorylation observed was not due to an increase in STAT3 and STAT1 protein levels, the density of the pSTAT3 and pSTAT1 band was normalized against total STAT3 and STAT1 proteins respectively, which remained constant across the samples. In contrast, pSTAT3 had decreased (n = 6, p<0.05) after 10 weeks of resistance training (TR10) compared to resting value (CTL10) (Fig. 1*a*). Moreover, immunofluorescence staining revealed that pSTAT3 co-localized with Pax7^+^ cells only at E2H (Fig. 1*b*), with no-detectable pSTAT3 at REST, E6H, CTL10 and TR10 indicating that STAT3 signaling was transiently active within SCs at E2H.

### Erk1/2 is phosphorylated in rat skeletal muscle following acute resistance exercise

**Figure 3:**
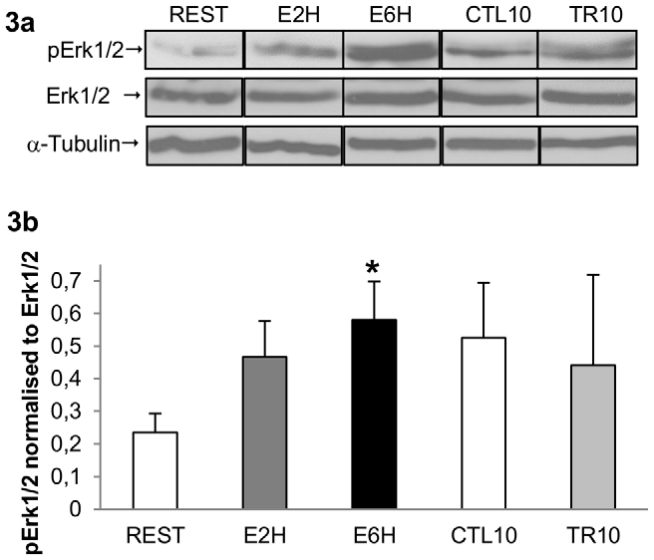
Erk1/2 activity in rat skeletal muscle following acute resistance exercise. ***A***: representative western blots of protein extracted from FDP muscle samples taken at REST, 2 hours (E2H) and 6 hours (E6H) after acute resistance exercise or after 10 weeks of resistance training (TR10) or rest (CTL10), with anti-phospho-Erk1/2 (Tyr202/204) (pErk1/2), anti-total Erk1/2 (tErk1/2) and α-tubulin. The arrow indicates the pErk1/2 bands at 42/44 kDa and α-tubulin at 55 kDa. ***B***: The graph shows arbitrary units of pErk1/2 normalized to tErk1/2 representing the mean ± SEM of 4–6 rats. * Significantly different from resting value (*p*<0.05).

Erk1/2 phosphorylation to total levels were significantly increased 6 hours after a single bout of exercise (E6H; Fig. 3) compared to resting values (REST, p<0.05). There was no change of Erk1/2 phosphorylation after 10 weeks of training (TR10) compared to resting values (CTL10) (Fig. 3)

### Satellite cell proliferation after acute resistance exercise

**Figure 4:**
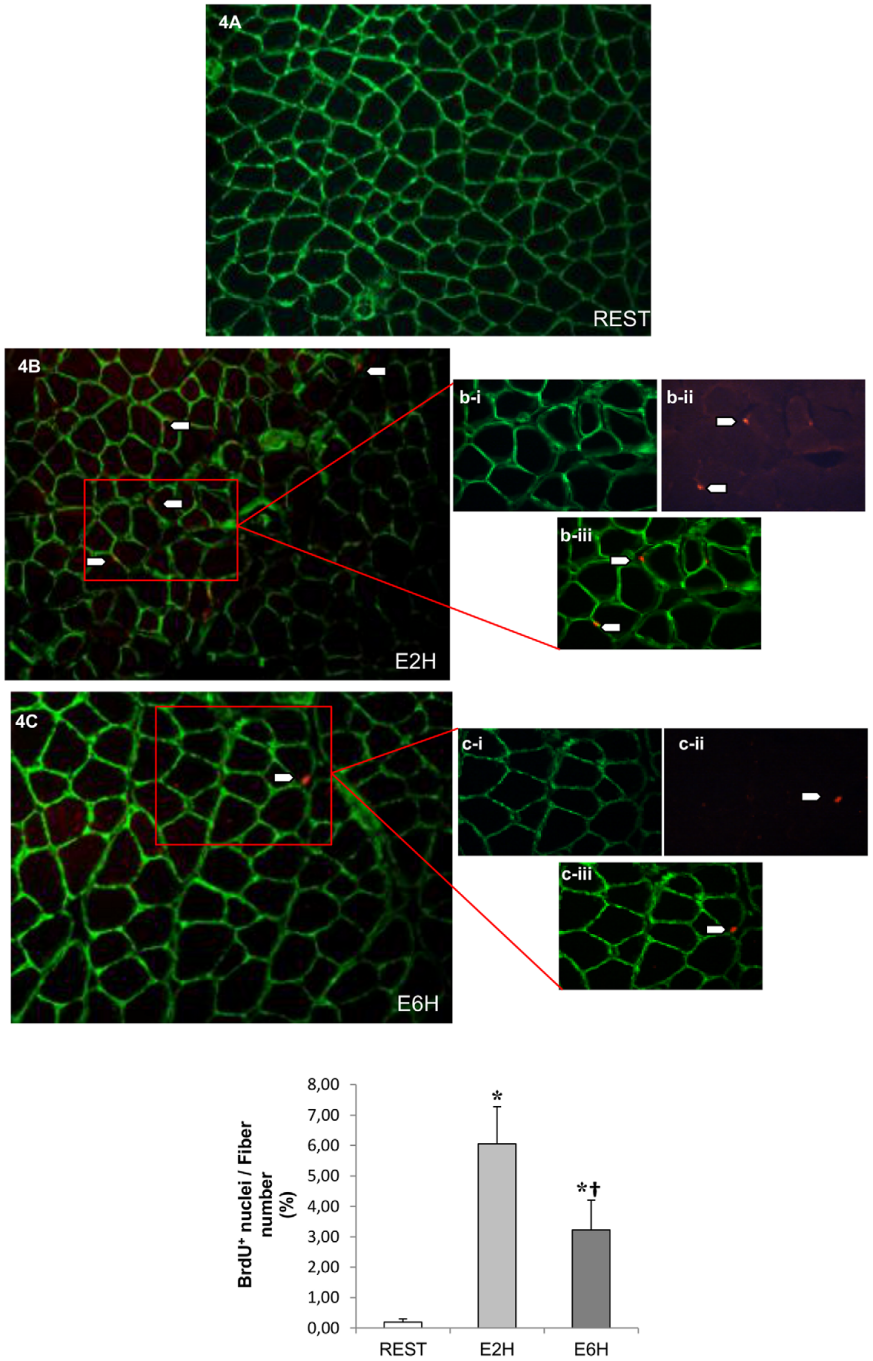
Satellite cell proliferation following acute resistance exercise. ***A–C***: Representative merged image (×20 magnification) of laminin staining (green) and BrdU-positive cells (red) of FDP muscle taken at REST (A), E2H (B), and E6H (C). **b–c**: Inset box (×40 magnification) of (**i**) laminin (green), (**ii**) BrdU (red), (**iii**) BrdU-positive satellite cells (merged image). ***D***: The graph shows the mean ±SEM of BrdU-positive satellite cells (%) expressed per 2000 fibers of 4 rats. * Significantly different from REST (p<0.05). † Significantly different from E2H (p<0.05).

To assess the involvement of SCs following acute resistance exercise, BrdU-positive cells situated between basal lamina and plasma membrane (intra-laminin staining) were counted to quantify satellite cell proliferating state (Fig. 4). When expressed in percentage of fibers, BrdU-positive SCs increased from 0.3% (REST) to 6%, 2 hours after exercise (E2H; p<0.05) and reached to 3%, 6 hours after exercise (E6H, p<0.05).Thus, resistance exercise contributes to activate SCs into proliferating state as early as 2 hours post-exercise.

### Impact of resistance exercise and training on STAT3-responsive genes

**Figure 5:**
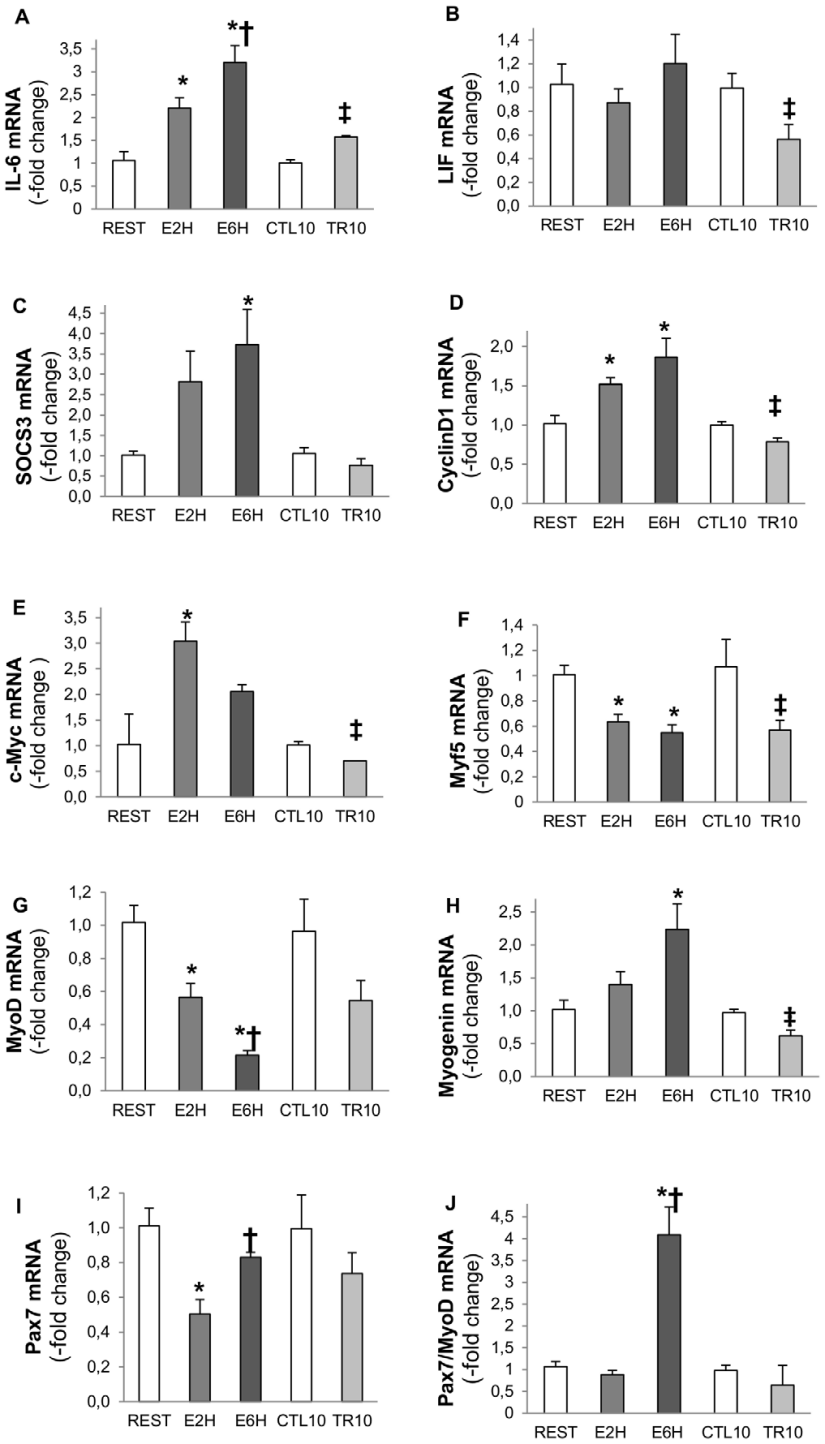
IL-6 (A), LIF (B), SOCS3 (C), CyclinD1 (D), c-Myc (E), Myf5 (F), MyoD (G), Myogenin (H), Pax7 (I), Pax7/MyoD (J) gene expressions. mRNA expressions 2 hours (E2H) and 6 hours (E6H) after a single bout of resistance exercise and 72 hours after 10 weeks of strength training program (CTL10, TR10) in FDP skeletal muscle of rats. Values are means of mRNA-fold change from REST or CTL10 ±SEM, n = 4–6 rats. * Significantly different from Rest (p<0.05). † Significantly different from E2H (p<0.05). ‡ Significantly different from CTL10 (p<0.05).

Downstream genes of STAT3 were investigated including IL-6, LIF, SOCS3, myogenic regulatory factors (MyoD, Myf5, Pax7, Myogenin) and markers of cell proliferation (CyclinD1, c-Myc) (Fig. 5).

### Myocyte mRNA expression of IL-6, LIF and SOCS3

The mRNA expression for IL-6 increased significantly 2 and 6 hours after a single bout of exercise, respectively 2.2-fold (n = 4, p<0.05) and 3.2-fold (n = 4, p<0.01), compared to resting values (Fig. 5*A*). No significant change was obtained for LIF mRNA expression after acute exercise (Fig. 5*B*). After 10 weeks of training, IL-6 mRNA expression also increased 1.57-fold (n = 6, p<0.01) compared to CTL10 values whereas LIF mRNA expression is downregulated (n = 6; p<0.05) (Fig. 5*A and B*). The mRNA expression for SOCS3 significantly increased 6 hours after a single bout of resistance exercise, 3.7-fold (n = 4, p<0.05), but tended to increase 2 hours after exercise (p = 0.08). No change was observed in SOCS3 mRNA expression after 10 weeks of resistance training (Fig. 5*C*). IL-6 and SOCS3 mRNA levels after a single bout of exercise are significantly correlated (R^2^ = 0.55, p<0.05) at 2 and 6 hours after exercise.

### mRNA expression of CyclinD1 and c-Myc are upregulated and correlate with IL-6 gene expression

The mRNA expression for CyclinD1 and c-Myc genes, two markers of cell proliferation, increased significantly in E2H group, respectively 1.5-fold (n = 4, p<0.05) and 3-fold (n = 4, p<0.05). CyclinD1 mRNA expression also increased 1.8-fold (n = 4, p<0.05) in E6H but it did not reach significance for c-Myc in E6H group (n = 4, p<0.05; Fig. 5 *D* and *E*). As for SOCS3, CyclinD1 mRNA expression correlated with IL-6 mRNA after a single bout of exercise (R^2^ = 0.64, p<0.05). On the other hand, significant decreases were observed in TR10 group for mRNA expression of CyclinD1 and c-Myc, respectively 0.78-fold (n = 6, p<0.05) and 0.70-fold (n = 6, p<0.05, Fig. 5 *D* and *E*).

### Myogenic Regulatory factor mRNA expression (Pax7, MyoD, Myf5, Myogenin)

The mRNA expression of Pax7, a marker of quiescent and activated satellite cells, MyoD and Myf5, markers of active satellite cell proliferation, significantly decreased 2 hours (E2H) after a single bout of exercise, respectively 0.50 (p<0.01), 0.56 (p<0.01) and 0.63-fold (p<0.01) (Fig. 5 *F*, *G and I*). MyoD and Myf5 mRNA also decreased 6 hours after the same exercise (E6H), respectively 0.21 (p<0.01) and 0.54-fold (p<0.01) whereas Myogenin mRNA increased 2.24-fold (p<0.05) (Fig. 5 *F*, *G* and *H*). No significant change in Pax7 and MyoD mRNA expressions was observed in TR10 group (Fig. 5 *G* and *I*), whereas Myf5 and Myogenin mRNA expressions significantly decreased in TR10 group respectively 0.56 and 0.62-fold (n = 6, p<0.05) (Fig. 3 *F, H*). The ratio Pax7/MyoD mRNA, a marker of satellite cell self-renewal, significantly increased in E6H, 4.08-fold (n = 4, p<0.01, Fig. 5*J*)

## Discussion

In the present study, we modified a physiological exercise model from Lee *et al.* (2002) [32] to induce hypertrophy and explored the link existing between the IL-6/STAT3 pathway and the acute and chronic SC activation through the myogenic regulatory factor (MRF) kinetic response. To our knowledge, no studies have focused on skeletal muscle hypertrophy after voluntary resistance training exercise in rats (i.e. without electric stimulation to force the animals, or compensatory hypertrophy with tonic-like acquired neuronal activity) [3], [35]. For example, the squat model apparatus by Tamaki *et al.* (1992) did not produce any skeletal muscle hypertrophy although the strength training was performed for 12 weeks (3 sets of 10 repetitions at 75% of 1 maximum repetition) [36]. With our physiological resistance training model, skeletal muscle hypertrophy occurred mainly after 10 weeks of resistance training in rats (Table 2). Similar to previous studies in humans [37], [38], we reported an increase in CSA for all fiber types but to a greater extent for type IIa (+92%) and type IIx (+100%) fibers in FDP muscle. It is well documented that resistance training could induce fiber hypertrophy through an enhancement of protein synthesis occurring just after the training session and lasting up to 24–48 h in humans [39], [40]. In parallel, depending on the exercise stimulus, the recruitment of additional nuclei derived from SC incorporated into muscle fibers could occur. Indeed, several works in humans have evidenced an increase in the number of myonuclei per fiber when fiber size increases approximately more than 25% [8], [41]. Thus, the myonuclear domain (i.e. the theoretical amount of cytoplasm supported by a single myonucleus in a muscle fiber) remained constant although a large increase in fiber CSA via the addition of SC-derived nuclei occurs. However, according to McCarthy *et al.* (2011), in a SC depleted mice muscle, a robust fiber hypertrophy can occur after synergist muscle ablation [42]. Such hypertrophy is associated with an expansion of myonuclear domain suggesting that SCs are not required to sustain hypertrophy in this model. Yet, in our study we found up to 100% of fiber size increase after 10 weeks of resistance training (TR10) which is associated to a constant myonuclear domain (Table 2), suggesting that in addition to the upregulation of protein synthesis, the SC population participates in fiber hypertrophy.

### Acute exercise

Serrano *et al.* (2008) have shown a blunted hypertrophic response in skeletal muscle of IL-6^−/−^ mice following compensatory hypertrophy, suggesting that IL-6 may play a role in skeletal muscle hypertrophy [18]. To further explore the link between the IL-6 pathway and the intervention of SCs in the hypertrophic response, our study has focused on MRFs and proliferating capacity through activation of the IL-6/STAT3 signaling pathway after resistance exercise in rats. Under specific conditions, the IL-6/STAT3 pathway could be relevant in SCs as it could mediate the hypertrophic response after resistance training in humans [20], [31]. In our animal model, we observed an increase in pSTAT3 at both 2 and 6 hours after resistance exercise (Fig. 1*a*), which was closely associated with an increase in IL-6, SOCS3, c-Myc and CyclinD1 mRNAs after a single bout of exercise (Fig. 5 *A*, *C*, *D* and *E*). One can argue that IL-6 gene up-regulation may come from local inflammation. However, we performed Hematoxiline/Eosine staining and did not find any presence of inflammatory cells (*data not shown*). The exercise-induced cytokine response differs from that in classical infectious context where TNF-α and IL-1β are the first secreted pro-inflammatory cytokines [43]. In humans, after exercise, TNF-α and IL-1β do not increase [44], [45] and IL-6 is usually the first cytokine present in the circulation [46]. There is now a growing evidence that acute exercise related IL-6 response act as an anti-inflammatory cytokine since IL-6 can exert inhibitory effects on TNF-α and IL-1 production [47] and stimulate the production of both anti-inflammatory cytokines IL-1ra and IL-10 [48]

STAT3 is activated in SCs in a transient manner as only pSTAT3 was detectable in SCs (Pax7^+^) after 2 hours of resistance exercise (Fig. 1*b*). Moreover, the number of mitotically BrdU positive SCs was significantly increased at both 2 and 6 hours after acute resistance exercise (Fig. 4) which is concomitant with the cell cycle markers CyclinD1 and c-Myc mRNAs (Fig. 5 *D* and *E*). These cells cycle markers are known as IL-6/STAT3-responding genes and have a critical role in cell growth and cell-cycle transition from G1 to S phase [49]. Altogether these results sustain IL-6 dependent SC proliferation. Others members of the IL-6 cytokine family, particularly the Leukemia Inhibitory Factor (LIF), could also contribute to STAT3 activation [50]. However, contrary to IL-6, the LIF gene stimulation was not significant 2 hours (E2H) or 6 hours (E6H) post-exercise in the present study (Fig. 5 *B*).

**Figure 6:**
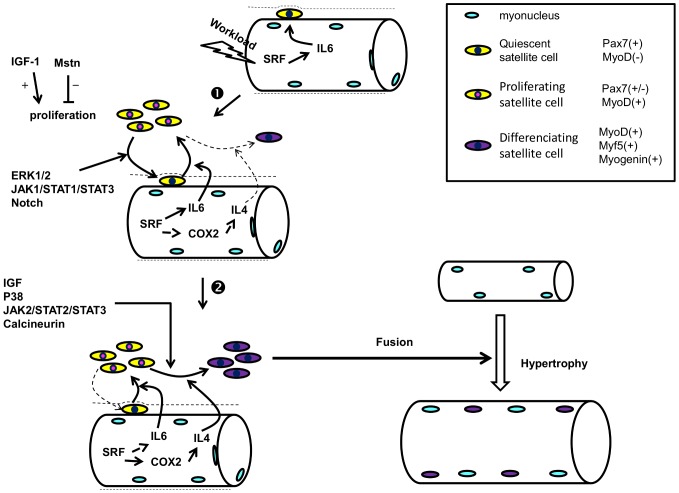
Involvement of IL-6/JAK/STAT pathway upon satellite cells behavior after resistance exercise and training. **1.** In response to increase workload, the SRF mediated IL-6 increasing production leads to satellite cells proliferation and first preferentially activate the JAK1/STAT1/STAT3 pathway leading to the rebuilt of the Pax7^(+)^/MyoD^(−)^ pool. Like the Notch activity for the maintenance of the Pax7^(+)^ SCs, the mitogen activated protein kinase Erk1/2 cooperates with the JAK1/STAT1/STAT3 pathway to repress early myogenic differentiation. **2.** Later, the decreased Notch activity, the activation of the JAK2/STAT2/STAT3 together with multiple pro-differentiating factors (p38, Calcineurin, IGF-1…) activate SCs which loose the Pax7 expression (Pax7^(−)^/MyoD^(+)^) and start to differentiate. At this stage, SCs express late MRFs (Myf5, Myogenin) and then fuse to existing myocytes leading to hypertrophy.

Most of the studies looking at MRFs both after injury in rodent and resistance training in humans [51], [52], suggested that the increase in MRF mRNA expression occurred at later time point (12 hours to 2 days). When focus is made on the early mRNA regulation of MRFs, we observed a downregulation of MyoD and Myf5 mRNAs, 2 and 6 hours after acute resistance exercise (Fig. 5 *F* and *G*) whereas an increase in Myogenin mRNA at 6 hours post-exercise was noted (Fig. 5 *H*). Similar results were obtained in humans by Costa *et al.* (2007) that reported a 45% decrease in MyoD mRNA, an absence of increase in Myf5 mRNA but an increase in Myogenin mRNA, 3 days after an eccentric training program [53]. Moreover, looking at the protein level, Sakuma *et al.* (1999) have also shown decreased plantaris MyoD content in the first 5–6 days after the ablation of both synergist soleus and gastrocnemius muscles, leading to compensatory plantaris hypertrophy in rats [54]. The significant up regulation of the Myogenin gene 6 hours (E6H) after acute resistance exercise suggests that some SCs are going to differentiate. As STAT1/STAT3 signaling pathway is early activated after our exercise, we first hypothesized that it could promote myoblast proliferation and prevent myoblast differentiation by inhibiting MyoD transcription [55]. The increased Myogenin mRNA shows that SCs differentiation is not completely abolished but suggests that different pool of SCs come into different states after exercise as suggested by Schultz et al. [56]. One part of SCs is specifying to become reserve cells as MyoD and Myf5 mRNAs are decreased along with the Pax7/MyoD ratio upregulation, but some SCs are able to engage differentiation early after exercise and fuse with existing myofibers to give their material. Thus, the SC population can be phenotypically and functionally divided into several compartments allowing different engagement. Thus, early after resistance exercise there might be a preferred proliferative phase of the first undifferentiated satellite cells in order to build reserve cells before engaging in a myogenic lineage, as suggested by Yoshida *et al.* (1998) [57] (Fig. 6). Precisely, this mechanism may result from activation of the JAK1/STAT1/STAT3 signaling pathway. Indeed, the STAT1/STAT3 signaling pathway activation could promote myoblast proliferation and prevent premature myoblast differentiation by inhibiting MyoD transcription [55] whereas the STAT2/STAT3 pathway is required for myogenic differentiation [25]. Interestingly, we showed a concomitant increase in pSTAT1, peaking at 2 h after exercise, and pSTAT3 at both 2 and 6 hours after exercise (Fig. 1*a*, 2; p<0.05). Thus, the early downregulation of MyoD and Myf5 mRNA could be mediated in part by the STAT1/STAT3 pathway in order to promote cell proliferation by STAT3 activation and repress cell differentiation via STAT1 [58], [59] (Fig. 6). This hypothesis is sustained by the significant upregulation of the Pax7/MyoD ratio observed at 6 hours after exercise (Fig. 5 *J*; p<0.01) suggesting that SCs return in a quiescent state. Moreover, the concomitant increase in pErk1/2 level (Fig. 3) could strengthen this proliferative phase, as the Erk1/2 pathway activation has been shown to inhibit differentiation at the early stage of differentiation but promote myocyte fusion in the late stage of differentiation [60]. As suggested by Fukuda *et al.* (1996) in pro-B cells lines [61], Erk1/2 phosphorylation at 6 hours after exercise (E6H, Fig. 3) may come from IL-6 signaling which is also required for proliferation of satellite cells mediated by STAT3 [25]. Finally, these data are in accordance with those of Sun *et al.* (2007) who pointed out a dual role of STAT1 and STAT3 in myoblast proliferation and differentiation [55].

### IL-6/STAT3 response to resistance training

After 10 weeks of resistance training, the IL-6 mRNA was still 1.4-fold higher than for the resting condition (Fig. 5 A). However, this increase was not accompanied by the upregulation of STAT3 target genes (SOCS3 mRNA) but instead by a downregulation of CyclinD1 and c-Myc mRNAs (p<0.05; Fig. 5 *C*, *D* and *E*). Accordingly, STAT3 was significantly less phosphorylated compared to the resting conditions (Fig. 1*a*). The decrease of pSTAT3 content could not be explained by an increase in the negative feedback loop initiated by the upregulation of SOCS3 since SOCS3 mRNA was not altered (Fig. 5 *C*).

Similarly to the results obtained with acute exercise, Myf5 (p<0.05) and MyoD (p = 0.1) mRNAs were reduced after resistance training and contrary to acute exercise also Myogenin mRNA was reduced (Fig. 5 *F*, *G*, *H*). Consistently, the decreased basal pSTAT3 content noted after 10 weeks of training (Fig. 1*a*) along with, CyclinD1, c-Myc, Myf5 and Myogenin mRNAs (Fig. 5 *D*, *E,F and H*) could be due to impairment in satellite cell proliferation. Previous studies showed that activated, proliferating satellite cells express both Pax7 and MyoD. Once activated, some cells then downregulate Pax7, maintain MyoD and differentiate, contrary to others which downregulate MyoD, maintain Pax7 expression and remain undifferentiated [62], [63] (Fig. 6). We suggest that the heavy resistance training proposed here may have acutely decreased the number of proliferative satellite cells in order to increase the quiescent satellite cell pool as the Pax7/MyoD mRNA ratio was increased 6 hours after exercise compared to the resting condition (Fig. 5 *J*). Moreover, the decreased basal pSTAT3 content after several weeks of heavy resistance training might be part of a protective mechanism from excessive muscle mass upregulation, as some fibers had already reached up to 100% hypertrophy As depicted recently by Chakkalakal et al. [63], it would be interesting to verify the Fgf2 signaling and/or sprouty1 (Spry1) expression in SC niche of resistance trained rats as an adaptive mechanism to limit hypertrophy.

In conclusion, the hypertrophic effect obtained after 10 weeks of resistance training in rat FDP muscle is acutely associated with the upregulation of the IL-6/STAT3 signaling pathway and the early downregulation of differentiating related MRF gene expressions. In fact, each acute resistance exercise bout in rat induces an increase in SCs IL-6 signaling through the activation of pSTAT3 and its dependent genes, CyclinD1 and c-Myc. The fast decrease in MRF mRNAs could reflect a proliferative phase of the satellite cell population mediated by STAT1/STAT3 activation in order to first rebuild the pool of reserve cells (Fig. 6). After 10 weeks of resistance training, the huge training-induced increase in muscle fiber cross-sectional area, up to 100%, is to be linked to the decrease of Pax7/MyoD mRNA ratio. This could be an adaptive mechanism to protect skeletal muscle from excessive hypertrophy.
